# Ursodeoxycholic Acid Attenuates the Retinal Vascular Abnormalities in Anti-PDGFR-β Antibody-Induced Pericyte Depletion Mouse Models

**DOI:** 10.1038/s41598-020-58039-x

**Published:** 2020-01-22

**Authors:** Tomoyasu Shiraya, Fumiyuki Araki, Takashi Ueta, Hisako Fukunaga, Kiyohito Totsuka, Takahiro Arai, Akiyoshi Uemura, Kyoji Moriya, Satoshi Kato

**Affiliations:** 1 Department of Ophthalmology, Graduate School of Medicine, The University of Tokyo, Tokyo, Japan; 2 Department of Infectious Diseases, The University of Tokyo Hospital, Tokyo, Japan; 3 Department of Retinal Vascular Biology, Nagoya City University Graduate School of Medical Sciences, Nagoya, Japan

**Keywords:** Retinal diseases, Experimental models of disease

## Abstract

As a clinical manifestations of diabetic retinopathy (DR), pericytes (PCs) loss from the capillary walls is thought to be an initial pathological change responsible for the breakdown of the blood-retinal barrier (BRB). This study was performed to investigate the effects of ursodeoxycholic acid (UDCA) in PC depletion mice by injection of an antibody against platelet-derived growth factor reception-β (PDGFR-β clone APB5). To assess the integrity of the retinal vessels, their density, diameters, vessel branching points, and number of acellular capillaries were evaluated. While all types of retinal vessels became enlarged in APB5-induced mice, treatment with UDCA rescued the vasculature; the vessel density, diameter of the veins and capillaries, and vessel branching points were significantly lower in mice treated with UDCA. Although APB5-induced mice displayed progressive exacerbation of retinal edema, whole retinal thickness upon treatment with UDCA was significantly decreased. Additionally, UDCA reduced the expression of F4/80^+^ macrophages in the APB5-induced retina according to immunofluorescent labeling. UDCA also reduced the increased expression of angiogenic factors and inflammatory mediators (vascular endothelial growth factor, intercellular adhesion molecule-1, and monocyte chemotactic protein-1). These findings suggest that UDCA can be used to prevent the progression of and treat DR.

## Introduction

Diabetes and its related complications, including retinopathy, have a great social and economic burden worldwide^1,2^. The World Health Organization has suggested that diabetic retinopathy (DR) is responsible for 15–17% of total blindness cases in the United States and Europe^1,3–5^. Pericytes (PC) not only provide mechanical support, but also maintain vessel wall integrity by interacting with endothelial cells (EC) via secretory signals and direct cell-to-cell contact^6^. As an early clinical manifestation of DR, PC depletion from the capillary wall is considered an early pathological change that causes the blood-retinal barrier (BRB) to collapse and subsequent vascular hyperpermeability^7^. Furthermore, PC depletion results in vascular abnormalities along with upregulation of angiogenic factors and inflammatory cytokines^8,9^. In cases of more advanced DR, vascular occlusion induces retinal hypo-perfusion and hypoxia, resulting in abnormal angiogenesis that directly causes blindness due to vitreous hemorrhage and tractional retinal detachment^10^. In recent years, intravitreal injections of anti-vascular endothelial growth factor (VEGF) agents have been effectively used to treat diabetic macular edema^11–13^ and have also empirically indicated the involvement of the VEGF signaling and inflammation in BRB breakdown in DR^14^. However, frequent injections are burdensome for patients and physicians, and are associated with a very high cost to the healthcare system. Preventative medicine of DR with a safe and low-burden treatment for patients that requires investigation.

Ursodeoxycholic acid (UDCA), a product of the hydrolysis of tauroursodeoxycholic acid, is now widely used in clinical applications such as for the treatment of primary biliary cirrhosis and various other cholestatic disorders^15^. The anti-inflammatory functions of UDCA have been reported^16,17^, such as suppressing eosinophilic inflammation^18^, inhibiting lipopolysaccharide-induced macrophages activation^19^. Also, it was found to attenuate these effects via inhibiting NFκB-mediated inflammatory signaling pathway^20^. Moreover, previous studies using streptozocin (STZ)-induced diabetic mice showed that UDCA attenuates endoplasmic reticulum stress-related retinal PC depletion^21^ and reduction the retinal expression of VEGF, restoration the BRB after breakdown based on a fluorescein permeation assay^20,21^.

To understand crucial processes occurring during a long-term course of DR, an animal model is needed in which direct sampling of specific cells or molecules can reproduce the characteristic features of DR. However, hyperglycemic animal models cannot fully imitate the pathophysiology of human DR. Therefore, the molecular and cellular mechanisms underlying barrier dysfunction of PC-free retinal vessels remain unknown^22^. This has made it difficult to determine whether the effect of UDCA listed above contributes to the development of late vascular changes observed in human diabetic retinas. PC deficiency was sufficient to reproduce the retinal vascular abnormalities characteristic of DR. Notably, PC recruitment was completely inhibited by administration of anti-platelet derived growth factor receptor (PDGFR)-β mAb (clone APB5) to neonatal mice^22,23^, in which the disease severity corresponded to the extent of PC depletion from developing retinal vessels, resulting in retinal collapse and BRB breakdown^22^.

In this study, we used an APB5-induced PC depletion model to investigate whether UDCA results in retinal vessel maintenance and inhibits inflammatory responses, thereby contributing to the attenuation of DR.

## Results

### UDCA attenuates the retinal vascular abnormalities in APB5-induced mice

**Figure 1:**
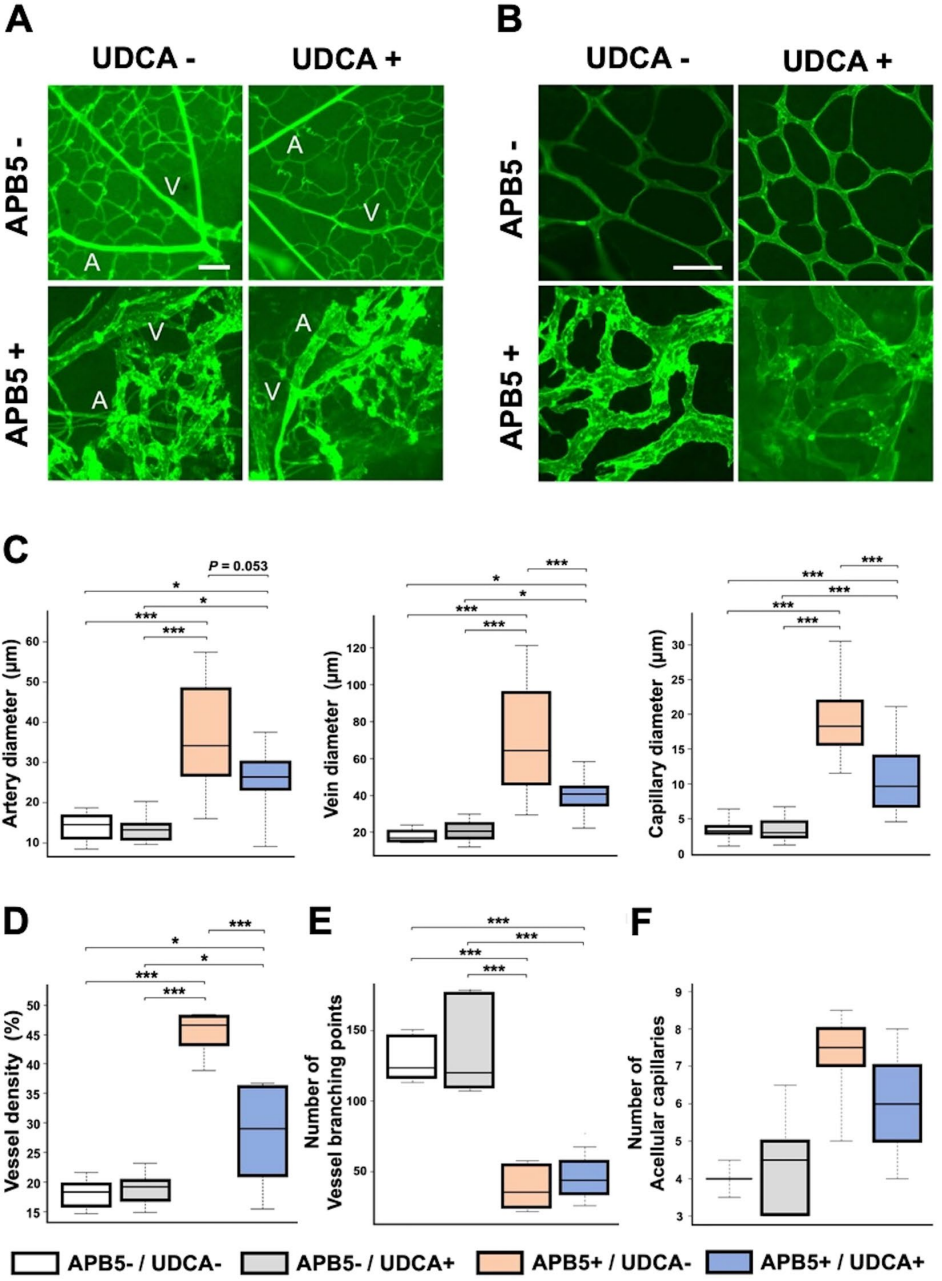
Ursodeoxycholic acid (UDCA) attenuates the retinal vascular abnormalities. Fluorescein-labeled concanavalin A was used for perfusion to obtain the angiographic images and to investigate the effect of UDCA on vessel morphology changes. Fundus images of the arteries and veins (**A**), capillary (**B**). A, artery; V, vein. (**C**) Quantification of vessel diameters of arteries (left), veins (middle), and capillaries (right). (**D**) The graph shows total vessel density (two 300 × 300 μm fields per retina). (**E**,**F**) Number of vessel branching points and the number of acellular capillaries in a unit area of 700 × 550 μm were measured. APB5−/UDCA− (n = 5); APB5−/UDCA+ (n = 6); APB5+/UDCA− (n = 6); APB5+/UDCA+ (n = 6). Data were expressed as the means ± standard error of the mean (S.E.M.). Asterisks indicate vessel lumen. *P < 0.05, **P < 0.01, ***P < 0.001 (one-way ANOVA). Scale bars: 100 μm (**A**); 50 μm (**B**).

In APB5-induced mice, all types of retinal vessels (arteries, veins, and capillaries) were enlarged and showed decreased vascular extensions; these vessels were anastomosed randomly with each other, as previously reported^23^. In contrast, enlargement of retinal vessels was suppressed by UDCA (Fig. 1A,B). The diameters of veins and capillaries were significantly lower than those in mice treated with UDCA in APB5-induced retinas (*P* < 0.001, *P* < 0.001, respectively) (F_3, 45_ = 25.73, *P* < 0.001, and F_3, 220_ = 284.2, *P* < 0.001, one-way ANOVA) (Fig. 1C). Although not significant, the artery diameter in the APB5+/UDCA+ group tended to be a lower than in the APB5+/UDCA− group (*P* = 0.053) (F_3, 45_ = 20.72, *P* < 0.001) (Fig. 1C). The vessel density was significantly lower in the APB5+/UDCA +group compared with the APB5+/UDCA− group (*P* < 0.001) (F_3, 20_ = 37.69, *P* < 0.001) (Fig. 1D). To further evaluate vascular changes, we also measured vessel branching points and the number of acellular capillaries in a unit area. The vessel branching points were significantly lower in the APB5-induced retinas than in non-APB5 induced retinas (*P* < 0.001) (F_3, 20_ = 29.42, *P* < 0.001) (Fig. 1E). However, there was no difference in the number of acellular capillaries among the two groups (*P* = 0.051) (F_3, 20_ = 3.42, *P* = 0.037) (Fig. 1F).

### UDCA attenuated retinal morphological changes in APB5-induced mice

**Figure 2:**
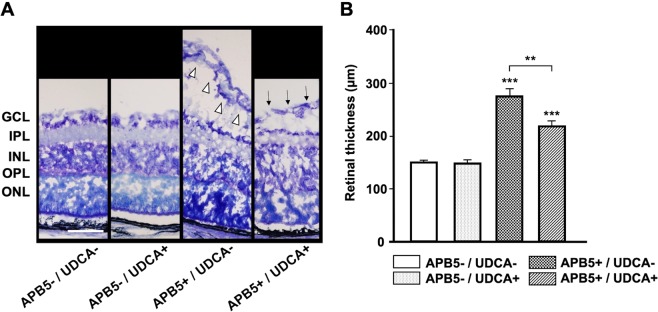
UDCA attenuated retinal morphological changes in APB5-induced PC depletion models. (**A**) Toluidine blue staining of retinas in each group. APB5-induced mice caused progressive exacerbation of retinal edema in GCL, and cells in the GCL displayed an irregular arrangement with an amplification of cell gaps (white arrowhead). In contrast, UDCA attenuated the increased retinal thickness of GCL (black arrow). Scale bar: 50 μm. (**B**) Quantification of the retinal thickness in each group. APB5−/UDCA− (n = 12); APB5−/UDCA+ (n = 13); APB5+/UDCA− (n = 12); APB5+/UDCA+ (n = 13). Data were expressed as the means ± standard error of the mean (S.E.M.). *P < 0.05, **P < 0.01, ***P < 0.001 (one-way ANOVA). GCL, ganglion cell layer; IPL, inner plexiform layer; INL, inner nuclear layer; ONL, outer nuclear layer.

As shown in Fig. 2A, in non-APB5-induced mice (APB5−/UDCA− and APB5−/UDCA+), retinal ganglion cells (GCLs), inner nuclear layers (INLs), and outer nuclear layers (ONLs) were all regularly arranged. UDCA had no effect on retinal morphology. In contrast, APB5-induced mice caused progressive exacerbation of retinal edema in GCLs, and GCL displayed an irregular arrangement with an amplification of cell gaps. UDCA attenuated the increased retinal thickness of GCL. Accordingly, whole retinal thickness in mice treated with UDCA was significantly decreased compared to those not treated with UDCA (*P* = 0.001) (F_3, 46_ = 35.92, *P* < 0.001, one-way ANOVA) (Fig. 2B).

### UDCA reduced the expression of F4/80^+^ macrophages in APB5-induced mice

**Figure 3:**
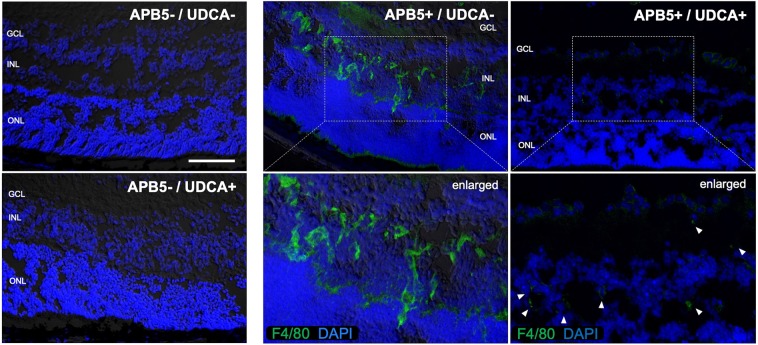
UDCA reduced the expression of F4/80+ macrophages in APB5-induced PC depletion mice. Immunofluorescent labeling with F4/80 (green) and DAPI (blue) is shown, and lower pictures are partial enlarged images. The number of F4/80+ macrophages decreased markedly with UDCA-treated mice in INL in the retina (white arrowhead indicate F4/80 immunofluorescent labeling). Scale bar: 100 μm. DAPI, 4–6-diamino-2-phenylindole.

The results of immunofluorescence staining assay in APB5-induced mice showed that F4/80^+^ macrophages were identified in INL in the retinas from non-UDCA-treated mice (APB5+/UDCA−). In contrast, the number of F4/80^+^ macrophages decreased markedly in UDCA-treated mice (APB5+/UDCA−) (Fig. 3).

### UDCA reduced the increased expression of VEGF, ICAM-1, and MCP-1 in retinas of APB5-induced mice

**Figure 4:**
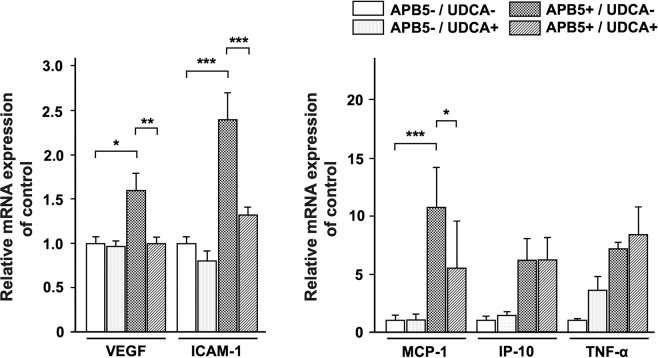
Retinal mRNA expression of angiogenic factors and inflammatory mediators. APB5 increased expression of VEGF, ICAM-1, and MCP-1, and these responses were attenuated by UDCA. UDCA reduced the increased expression of VEGF, ICAM-1, and MCP-1 from APB5-induced mice (P = 0.004, P < 0.001, and P = 0.007, respectively). APB5−/UDCA− (n = 5); APB5−/UDCA+ (n = 8); APB5+/UDCA− (n = 8); APB5+/UDCA+ (n = 9). Data were expressed as the means ± standard error of the mean (S.E.M.). *P < 0.05, **P < 0.01, ***P < 0.001 (one-way ANOVA).

As shown in Fig. 4, the APB5+/UDCA− group displayed an increased mRNA expression by quantitative polymerase chain reaction (qPCR) analysis of VEGF, intercellular cell adhesion molecule (ICAM)-1, and monocyte chemotactic protein (MCP)-1 compared to the APB5−/UDCA− group (F_3, 26_ = 7.14, *P* = 0.001, and F_3, 26_ = 15.97, *P* < 0.001, and F_3, 24_ = 20.93, *P* < 0.001, respectively, one-way ANOVA). The expression of interferon-inducible protein (IP)-10, and tumor necrosis factor (TNF)-α showed no significant difference between the APB5+/UDCA− group and APB5−/UDCA− group (*P* = 0.23 and 0.74) (F_3, 26_ = 3.23, *P* = 0.039; F_3,21_ = 2.07, *P* = 0.14, respectively). Importantly, UDCA reduced the increased expression of VEGF from APB5-induced mice (*P* = 0.004). Moreover, UDCA also reduced the increased expression of ICAM-1 and MCP-1 from APB5− induced mice (*P* < 0.001, *P* = 0.007, respectively).

## Discussion

PCs maintain the integrity of blood vessel walls and play pivotal roles in controlling vascular development and homeostasis. In the APB5-injected retina, PCs were often partially or completely dissociated from ECs^22^. Because of transient failure of PC recruitment into the growing retinal vessels, irreversible defects occurred in EC-PC associations, resulting in BRB breakdown^22^. In this study, we adopted a mouse model with depleted PC in retinal vessels by systemically injecting postnatal mice with a monoclonal antibody for PDGFR-β (clone APB5). Our model improves on previous attempts, as hyperglycemic animal models such as STZ-induced mice model fail to fully mimic the pathophysiology of human DR, particularly in terms of retinal vascular changes. Our study evaluated the effect of UDCA on retinal vessel maintenance and the inhibition of an inflammatory response using APB5-injected mice. We identified the effects of UDCA using angiographic images with fluorescein-labeled concanavalin A, and observed retinal morphological changes, expression of F4/80^+^ macrophages into the retinas, and changes in the mRNA expression of angiogenic factors and inflammatory mediators. These results indicate that UDCA attenuates the retinal vascular abnormalities and retinal morphological changes. Furthermore, reduced expression of F4/80^+^ macrophages and a reduction the increased expression of VEGF, ICAM-1, and MCP-1 were observed in the retinas.

In mice, retinal vasculature development occurs in a similar to that in humans, but begins postnatally^24^. In mouse retina, at postnatal day 1 (P1), a primitive vascular plexus begins to sprout radially from the optic disc located in the center of the retina. As vessels spread toward the periphery, they reach the edges of the retina at P8-P10^22,25^, and then sprout downward at approximately P7 to form deep and intermediate vascular layers. In this process, ECs at the tips of the sprouting vessels express PDGF-B, thereby promoting the growth and migration of PCs that express PDGFR-β along the emergent vessels^22,25^. Consequently, most of the ECs associate with PCs over the developing retinal vasculature. Therefore, administration of anti- PDGFR-β mAb to neonatal mice can inhibit PC accumulation during retinal blood vessel development, enabling reproduction of the same vascular abnormalities as in DR^23^.

In our study, UDCA injection was performed daily from P7 through to P9, and the effects were investigated in P10 retinas. Doses and injection timings were based on the following results of previous experiments and the following: in APB-5 treated retinas, infiltration of perivascular macrophages at P6 is increased^22^; in P8 retinas, pro-inflammatory genes such as TNF, IL-6, and MCP-1 were continuously upregulated in whole retinas^22^, which showed, increased infiltration of leukocytes and endothelial upregulation of ICAM-1, progressive vascular leakage and hemorrhage^22^. Finally, the status of APB5-induced retinal collapse (40 μg) did not change at later stages, indicating P10 as a turning point^22^.

As previously reported, in the APB5-treated retinas, all types of retinal vessels were enlarged, with decreased vascular extensions and increased vessel densities^22,23^, and the ECs lost their spindle shapes and were disarranged in all types of vessels. Furthermore, although preformed intravascular PC-free ECs maintained high mitotic activities, they failed to sprout downward to the deep retinal layers. Consequently, the formation of aneurysm-like bodies leading to EC apoptosis occurred^22^. Together, these findings suggested that the ECs were continuously sensitive to the microenvironments in the absence of PCs, thereby causing dysregulated vascular remodeling^22^. In our study, UDCA attenuated the retinal vascular abnormalities: vessel density, which is the diameter of veins and capillaries, was significantly lower. The arteries showed a similar tendency. These results suggest that UDCA restored the dysregulated remodeling of these vessels.

PC depletion directly induced inflammatory responses in ECs and the perivascular infiltration of macrophages and these actions caused vessel damage via VEGF, placental growth factor, and angiopoietin-2^22^. Expression of F4/80 is heterogeneous and is modulated during macrophage maturation and activation. The results of the F4/80 immunofluorescence staining assay showed that UDCA reduced the expression of F4/80^+^ macrophages in APB5-induced retinas. The recruitment of macrophages is an important marker of the inflammatory response. Therefore, our results suggest that the anti-inflammatory function of UDCA suppressed the migration of macrophages into the retina.

Various studies have shown that not only anti-VEGF therapy, but also anti-inflammatory treatment is an effective strategy for DR treatment^26,27^. In PC depleted retinas, expression of VEGF is upregulated under hypoxia conditions because of impaired blood flow^22^. Our results showed that UDCA reduced the mRNA expression of VEGF in the APB5-induced retina. The following may be responsible for this desirable effect: UDCA attenuated vascular destruction based on our findings and restored BRB breakdown by abrogating the NFκB-mediated inflammatory signaling pathway^20^, which inhibited the progression of retinal ischemia and therefore suppressed VEGF expression; UDCA suppressed macrophage-derived VEGF according to our result of immunostaining results. Moreover, in terms of the action mechanism of UDCA, a previous study showed that tauroursodeoxycholic acid (formed by the conjugation of UDCA with taurine) suppressed the increased expression of VEGF in high glucose-induced retinal microvascular endothelial cells^28^, demonstrating that UDCA also functions targeting the vascular endothelium.

Although APB5-induced mice showed progressive exacerbation of retinal edema, UDCA attenuated the increased retinal thickness, which can also be explained by decreased VEGF and the anti-inflammatory effect caused by UDCA observed in qPCR. Furthermore, in a previous study, APB5-induced retinas demonstrated increased infiltration of leukocytes and endothelial upregulation of ICAM-1^22^. Leukostasis occurs early in the DR process. Increased leukostasis leads to upregulation of ICAM-1 and retinal vascular leakage^29^. Our qPCR results also revealed a decrease in ICAM-1.

There were some limitations to our study. Although the PC depletion mouse model using APB5 effectively reproduces the retinal vascular abnormalities characteristic of DR, pathological changes are rapid and retinal collapse can occur even in a pup mouse. In this study, our models were treated with UDCA at a very early stage (P7-P9) to enable assessment of sequential events due to retinal collapse. However, in DR, PCs loss from capillary walls were chronically progressed over the many years. Additional studies are needed to determine whether similar effects can be obtained by UDCA for various pathological changes over a long period of time in patients with DR.

In conclusion, UDCA attenuates retinal vascular abnormalities by rescuing dysregulated remodeling through the suppression of VEGF and an inhibition of inflammation. Therefore, UDCA represents a potentially impactful treatment for the prevention or treatment of progressive DR.

## Methods

### Experimental animals

All animal experiments followed the Association for Research in Vision and Ophthalmology guidelines and were approved by the Institutional Animal Care and Use Committee of the University of Tokyo (Tokyo, Japan [approval number M-P18-04]. Specific pathogen-free C57BL/6 J pregnant mice were purchased from CLEA Japan, Inc. (Tokyo, Japan). The mice were fed a standard laboratory diet and given free access to tap water, and lived in a room with controlled temperature and humidity, with a 12:12-h light: dark cycle. This experiment was initiated by injecting postnatal mice with a rat anti–mouse PDGFR-β mAb (clone APB5)^23^. In this study, 50 μg of APB5 dissolved in 0.05 mL of phosphate-buffered saline (PBS) was intraperitoneally injected once at P1. This dose of APB5 was selected based on experiments by Ogura *et al*.^22^. For controls, the same volume of PBS was intraperitoneally injected. Next, subcutaneous injection was performed in daily for P7-9 neonates with UDCA (Wako, Osaka, Japan; 100 mg/kg) in NaHCO3 (1.5 mol/L) or the same volume of NaHCO3 (as a control). The dose decision was also based on the previous experiments^30^. All experimental procedures in this study investigated the mice at P10. The treated mice were classified into four groups: APB5−/UDCA−, APB5−/UDCA+, APB5+/UDCA−, and APB5+/UDCA+.

### Morphological analyses: retinal vessel integrity

Fluorescein labeled concanavalin A (Con A; Vector Labs, Burlingame, CA, USA) was used for perfusion to label vascular ECs and to obtain the angiographic images as previously described^31^. Mice were anesthetized and their chests were opened to expose the heart; the right atrium was cut for drainage, a 27-gauge cannula was inserted into left ventricle, and then perfused over a span of 1 minute with Con A (0.5 mL at 625 μg/mL in PBS). Eyes were removed and fixed in 4% paraformaldehyde (PFA) for 1 h at room temperature, and retinas were dissected and flat-mounted. Images were acquired using a fluorescence microscope (BZ-9000, Keyence, Osaka, Japan). The vessel density was calculated as the proportions of Con A staining, in two 300 × 300 μm fields per retina. Accordingly, the artery and vein diameters were measured by averaging the values at 4–5 points per vessel, and the capillary diameter was also measured in 20 capillary branches per retina. Moreover, the number of vessel branching points and the number of acellular capillaries in a unit area of 700 × 550 μm were measured. Images were analyzed using NIH ImageJ software (version 1.52: Bethesda, MD, USA).

### Retinal histological evaluation

Eyes were enucleated under anesthesia and were fixed in 4% PFA in PBS for 1 h at room temperature. Next, the eyes were frozen in optimum cutting temperature (OCT) compound (Tissue-Tek; Sakura Finetek, Thatcham, UK) and sectioned at a thickness of 10μm and then thaw-mounted onto glass slides. The specimens were dried for 30 min at room temperature, rehydrated in PBS for 5 min, and stained with toluidine blue (WALDECK GmbH & Co KG, Münster, Germany), after which they were photographed under a microscope (DP74, Olympus, Tokyo, Japan).

### Immunohistochemical analysis

Frozen 10-μm sections on slides were dried for 30 min at room temperature, rehydrated in PBS for 5 min, incubated with blocking solution (5% normal goat serum and 0.5% Triton X-100 in PBS) for 1 h, and then stained with primary antibodies for overnight at 4 °C overnight. The slides were washed with PBS three times for 10 min each time and incubated with secondary antibodies at 4 °C overnight^32^. We used a rat monoclonal antibody specific to F4/80 (Cl: A3-1, 1: 1000, Bio-Rad Laboratories, Inc. Hercules, CA, USA) for the primary antibody and an Alexa Fluor 488-conjugated secondary antibody (1:400), and the nuclei were stained with DAPI (1: 2000). The specimens were observed under a laser confocal microscope (LSM800, Zeiss, Oberkochen, Germany).

### Quantitative RT-PCR

Real-time polymerase chain reaction (RT-PCR) analysis was carried out as follows. Total RNA in the retinas was isolated by using Trizol reagent, and the RNA content was determined. cDNA was synthesized according to the manufacturer’s instructions. RT-PCR was performed using kits, and the relative expression of target genes was normalized to GAPDH, analyzed by the 2^-ΔΔCt^ method and given as a ratio compared with the control^20^. The following primers were used for the analyses: VEGF forward (GCCAGCACATAGGAGAGATGAGC), VEGF reverse (CAAGGCTCACAGTGATTTTCTGG); ICAM-1 forward (GGCACCCAGCAGAAGTTGTT), ICAM-1 reverse (CCTCAGTCACCTCTACCAAG); IP-10 forward (CAGTGAGAATGAGGGCCATAGG), IP-10 reverse (CTCAACACGTGGGCAGGAT); MCP-1 forward (ATTGGGATCATCTTGCTGGT), MCP-1 reverse (CCTGCTGTTCACAGTTGCC); TNF-α forward (CATGAGCACAGAAAGCATGATCCG), and TNF-α reverse (AAGCAGGAATGAGAAGAGGCTGAG).

### Statistical analysis

Date were expressed as the means ± standard error of the mean (S.E.M.). Statistical significance was determined by one-way analysis of variance (ANOVA), followed by Bonferroni’s post-hoc tests, as indicated in the figure legends. F value and degrees of freedom were also provided in the statistical results. *P* values < 0.05 were considered as significant. All statistical analyses were performed using R version 3.5.2.^33^.

## Data Availability

All data generated or analyzed during this study are included in this published article.
